# The Observation of Pediatric Skull Fractures Without an Associated Brain Injury in a Non-Trauma Center

**DOI:** 10.7759/cureus.50571

**Published:** 2023-12-15

**Authors:** Muhammad Waseem, Kathryn D Esposito, Katherine Cedano, Masood A Shariff, Soula Priovolos

**Affiliations:** 1 Emergency Medicine, New York City (NYC) Health + Hospitals/Lincoln, New York, USA; 2 Surgery, Maimonides Medical Center, Brooklyn, USA; 3 Pediatrics, New York City (NYC) Health + Hospitals/Lincoln, New York, USA; 4 Internal Medicine, New York City (NYC) Health + Hospitals/Lincoln, New York, USA; 5 Surgery, New York City (NYC) Health + Hospitals/Lincoln, New York, USA

**Keywords:** trauma, pediatric emergency medicine, skull fracture, emergency medicine, pediatric head trauma

## Abstract

Introduction

Young children experiencing head trauma are prone to skull fractures. Pediatric skull fractures are distinct from adults as they have a greater capacity to undergo remodeling. The objective of this study was to evaluate whether children with isolated skull fractures without an underlying brain injury and normal neurological exam require a transfer to a tertiary hospital with pediatric neurosurgery service.

Methods

A retrospective chart review was performed to review children under five years old presenting to the emergency department of a non-pediatric trauma center with an isolated skull fracture resulting from head trauma without intracerebral hemorrhage between 2015 and 2021. The inclusion criteria consisted of children who have isolated skull fractures without underlying injuries and normal neurological examination.^ ^We reviewed these patients' injury characteristics, disposition, and clinical outcomes. The t-test and chi-square were used for evaluating the groups and evaluating the transfer to a dedicated trauma care facility.

Results

We identified 26 children who had isolated skull fractures with no underlying brain injury and normal neurological examination. The two most common mechanisms of injury were falls (64%) and motor vehicle collisions (MVC) (11%). The median age of patients was six months old. The location of the skull fractures was as follows: parietal (46%), occipital (19%), temporal (15%), frontal (7.7%), occipital + parietal (7.7%), and parietal + frontal (3.8%). Four fractures were depressed (15%) and the remainder were non-displaced. Eleven children with skull fractures (42%) were transferred to a designated pediatric trauma center and the remaining 58% were hospitalized for observation and monitored at the primary hospital. None of the children with skull fractures required intubation or other advanced interventions.

Conclusion

In this relatively limited sample, approximately one-third of the children with isolated skull fractures without brain injury were managed successfully in a non-tertiary care center. However, none of them required surgical intervention. Thus, we propose that patients akin to those in this study can be observed at a local hospital without being transferred to a pediatric trauma center.

## Introduction

Children experiencing head trauma are particularly prone to skull fractures. Skull fractures in the pediatric population cause morbidity and mortality [1-4]. Isolated skull fractures are commonly seen in injuries in the Emergency Department (ED) [5]. Young children with isolated skull fractures are often hospitalized for neurologic monitoring and observation. Despite the commonality of skull fractures, serious complications and neurosurgical interventions are rare [1-2]. It has been reported that less than one percent of skull fractures require neurosurgical intervention. More efforts are being made to determine if these children can be sent home, avoiding unnecessary transfers, hospital admissions, and associated costs [3,5-7]. The purpose of this study is to describe the injury characteristics and clinical outcomes in children with isolated skull fractures.

## Materials and methods

After institutional review board approval (FWA#00009807), we screened all patients, 0 to 5 years of age, who presented to the ED between the 1st of January 2015 and the 30th of December 2021, and we reviewed their medical records all patients with the following characteristics to screen them for head trauma in an inner-city hospital pediatric ED in the borough of South Bronx in New York City. This study occurred at the facility, a state-designated level 1 adult trauma center with massive transfusion capability and an onsite pediatric intensive care unit (PICU), but without an onsite pediatric surgery and neurosurgery service. The hospital serves a low socioeconomic urban minority population. The inclusion criteria included children with head trauma with isolated skull fractures and had a normal neurological examination. The exclusion criteria were evidence of intracerebral hemorrhage and abnormal neurological examination.

From chart review, we examined the patients’ demographics, mechanisms of injury, physical findings, imaging studies, fracture location (displacement/non-displacement), PICU admissions, and treatments and interventions (if any). We also reviewed their disposition, transfer decision, and length of stay in the ED. Statistically, t-test and chi-square analysis were used for evaluating the differences.

The pediatric ED follows a systematic and multidisciplinary team approach for the evaluation, such as the PECARN (Pediatric Emergency Care Applied Research Network) Head Trauma Protocol, a clinical guideline designed to assist healthcare providers in assessing and managing head injuries in children [8]. Table 1 lists the diagnostic criteria used for discharge. The patients in the observation group were provided an appointment at follow-up with a pediatric primary care provider (PCP) and trauma service.

**Table 1:** Discharge criteria for non-displaced, isolated skull fracture pediatric patients

No significant extracranial injuries
No indications of intracranial injuries
Normal neurological examination
Ability to arouse easily
No concern for child abuse
Residence in close proximity to the hospital
Reliable caretakers who can return with the child if necessary

## Results

We identified 26 children with isolated skull fractures and normal neurological examination (Table 2).

**Table 2:** Patient characteristics Continuous variables are presented as mean ± standard deviation and categorical as n and frequency, n (%). *MVC = motor vehicle collision ** Hemorrhage noted on the CT scan imaging: subdural, subarachnoid, and epidural.

Variables	Fracture
N	26
Age (years)	1.0±1.3
Median, years	0.52 (0.27-0.76)
Gender	
Male	12 (46.2%)
Female	14 (53.8%)
Length of stay (days)	3.1±1.6
Race	
African American	3 (11.5%)
Hispanic	12 (46.2%)
Others	11 (42.3%)
Radiology	
CT Scan, only	25 (96.2%)
X-rayand CT scan	1 (3.8%)
Injury Mechanism	
Fall	16 (61.5%)
Abuse	1 (3.8%)
Unknown	5 (19.2%)
MVC*	1 (3.8%)
Dog bite	1 (3.8%)
Sledding accident	1 (3.8%)
Abuse/Fall	1 (3.8%)
Associated Injuries	
Abdominal injury	1 (3.8%)
Hematoma-Facial/Scalp	8 (30.8%)
Hemorrhage**	6 (23.1%)

The average age of children presenting to the ED with overall head trauma (both with and without skull fracture) was 1.0±1.3 years old. In the 26 patients with skull fractures, the median age was six months old. Of those with isolated skull fracture(s), 46% were male. Demographically, 46% identified as Hispanic, 12% Black, and 42% “other”. Most patients with head trauma, regardless of fracture status, were brought into the ED by private vehicle (71%), followed by Emergency Medical Services (EMS) (17%), and others (12%).

The mechanisms of injury from most prominent to least were falls (61.5%), unknown (19.2%), motor vehicle collisions (MVC) (n=1), dog bite (n=1), and sledding accidents (n=1). The most common mechanism of injury was a fall in this dataset.

Fracture characteristics such as fracture location and description were also studied (Table 3).

**Table 3:** Characteristics of patients with isolated skull fracture PICU = pediatric intensive care unit

N	26
Fracture Location	
Frontal	2 (7.7%)
Parietal	12 (46.2%)
Temporal	4 (15.4%)
Occipital	5 (19.2%)
Parietal/Frontal	1 (3.8%)
Occipital/Parietal	2 (7.7%)
Fracture Description	
Depressed	4 (15.4%)
Non-displaced	22 (84.6%)
Disposition	
Observation at Primary Hospital	
PICU	9 (34.6%)
lnpatient Service	6 (23.1%)
Transferred to Level 1 Pediatric Trauma Center	11 (42.3%)

The location of the fractures varied; these include parietal (46%), occipital (19%), temporal (15%), frontal (7.7%), occipital + parietal (7.7%), and parietal + frontal (3.8%) regions. Four fractures were depressed (15%), and the remainder were non-displaced (n=22). Additional associated injuries in the cohort included hematomas of the face and scalp and abdominal bruising.

In our cohort (n=26), 11 children (42.3%) were transferred to a designated tertiary care pediatric trauma center from our ED, and 15 (57.7%) were hospitalized and monitored at our primary hospital. Of those that required transfer, CT-head findings were significant for subdural (3/11; 27%), subarachnoid (2/11; 18%) and epidural hemorrhage (2/11; 18%), scalp hematoma at the site of fracture (2/11; 18%). Two (18%) patients required transfer based on physician discretion. The patients in our cohort that stayed in our facility for observation only (n=15) had an average length of stay of 3.1 days (range 1 to 6 days). Of those patients that were admitted for observation, nine patients (35%) were admitted to the general pediatric inpatient service, and six (23%) were sent to PICU for closer observation. All the hospitalized children had a Glasgow Coma Scale (GCS) of 15 on arrival. None of the children in the cohort required intubation or other advanced interventions.

The results of this study were previously presented as a meeting abstract at the 2022 American College of Emergency Physicians Research Forum on October 1-4, 2022.

## Discussion

In the pediatric population, head trauma often results in skull fractures. Pediatric skull fractures require age-specific treatment and should not have the same treatment plan as adult fractures. Unlike their adult, the pediatric skull has a greater capacity to remodel; concurrently, pediatric brains are still developing [9]. Though much research has been conducted studying pediatric trauma, literature is sparse in terms of isolated skull fractures. Our study aimed to help fill this knowledge gap by determining whether it is necessary to transfer pediatric patients with isolated skull fractures to tertiary centers for neurosurgical evaluation or if they could be closely observed at the primary care center.

﻿The PECARN Head Trauma Protocol is a clinical guideline designed to assist healthcare providers in assessing and managing head injuries in children [8]. It considers age-specific criteria, GCS score, and the duration of loss of consciousness (LOC) to evaluate the severity of head injuries in children systematically. This protocol stratifies patients into low-, intermediate-, or high-risk categories based on their clinical presentation, helping healthcare providers make informed decisions about the need for CT scans. For low-risk patients, the protocol discourages unnecessary CT scans to minimize radiation exposure, reducing unnecessary radiation exposure while ensuring the timely detection of serious injuries, whereas intermediate and high-risk patients receive clear indications for CT imaging [8,10], in addition to the emphasis on parental education. PECARN prioritizes patient safety, minimizes radiation exposure, and ensures timely identification and treatment of serious head injuries while providing a structured risk assessment and management framework. Although this protocol stratifies the imaging a patient should receive, it does not delineate disposition criteria for admission versus observation.

A common practice is to admit children with skull fractures to the hospital for observation. Neurologically intact children with an isolated skull fracture without intracranial hemorrhage do not require neurosurgical intervention. However, patients with worrisome findings may be referred to tertiary hospitals with pediatric neurosurgery capabilities. Recently, more efforts have been made to reduce unnecessary hospitalizations. Studies suggest that children with linear non-displaced skull fractures and no intracranial hematoma after head trauma have a very low risk of evolving other traumatic findings or requiring neurosurgical intervention, so observation in the ED may be sufficient [11]. Overall, there is no consensus on the appropriate course of action in children with isolated skull fractures, so there is considerable variability in the standard of care. In our cohort, there were 26 patients found to have a fracture via a CT scan of the head. Among these patients, 11 were transferred to another tertiary care facility. These individuals were noted to have hemorrhage in the epidural, subdural, and subarachnoid regions, and some had a hematoma along the fracture line. The pediatric ED physician team and clinical exam determination delineated the transfer of these patients. The remaining 15 were admitted and observed in the Inpatient and PICU services.

A recent study by Barba et al. found that multi-level falls (MLF) accounted for upwards of 37.7% of pediatric basilar skull fractures [12]. Perheentupa et al. found that the most common skull base fracture type was the temporal bone fracture (64%), with road traffic accidents as the primary etiology [13]. Leibu et al. also found the temporal bone to be the most common fracture location (57%) but via falls [14]. In our ED, most skull fractures were of the parietal bone (46%), and the most common etiology for isolated skull fractures was from falls (62%). There is much variability in fracture location and etiology in children.

There is no universal protocol in place for the standard of care regarding the decision to admit for observation versus transfer versus discharge of a patient following a head trauma encounter with an isolated skull fracture in the ED, and often, these patients are admitted for observation. Frequently, neurologically normal children who have an isolated (basilar) skull fracture without any intracranial hemorrhage do not require any neurosurgical intervention [9]. Since there is no consensus on the appropriate course of action in children with isolated skull fractures, there is considerable variability in their evaluation. In this study, all patients received head CT as part of the generalized trauma protocol. However, Barba et al. suggest that CT examinations only detected abnormalities in 1.9% of patients, so it is hard to know if CT scans are, in fact, necessary for every head trauma [8,12]. Head CT is preferred over simple radiography due to the ability to dual identify skull fractures and traumatic brain injury. The threshold for obtaining a CT scan in infants younger than two years, particularly those younger than three months, should be lower than for older children [4,10,15,16]. Skull radiographs may be performed when trauma history is uncertain [14]. After the X-ray, one patient was directed to the CT suite for a head scan at the physician's discretion.

Powell et al. described a set of criteria for admitting pediatric skull fracture patients, but this is not the universal gold standard. Signs of increased intracranial pressure, such as persistent neurologic deficits, headache, or vomiting, intracranial injury, suspected child abuse, and parents or caregivers who are unreliable or unable to return, if necessary, require an admission [17]. Additionally, those patients with depressed skull fractures (15% in our study) do require neurosurgery consultation [17]. This process could be protocolized to evaluate and follow up with the patient during admission to streamline recommendations with discharge and outpatient services guidance.

Children with isolated skull fractures, specifically those that are non-displaced/depressed, which accounted for 85% of our patient population that is neurologically intact, can be safely discharged home if specific discharge criteria are met [16]. Patients are instructed to follow up with their pediatric PCP within one to two days of injury, which also brings up a similar issue; the child might not have a PCP, or their caretaker might not be able to take another day off from work [16]. Our patients may not fit the proposed criteria for admission, but they may still get admitted due to their social determinants of health [16,18]. It is possible that different protocols need to be in place as a contingency plan if adequate follow-up is not available.

Though our study has limitations, including its retrospective study design with a relatively small sample size and conducted in a low socioeconomic patient population, the results of our study demonstrate no advantage in hospitalization of children with isolated skull fractures who otherwise have normal neurologic examination as no clinical deterioration noted during observation. Few patients were noted as having an unclear mechanism of injury. This is a limitation as the chart was reviewed to explore if abuse was at play, and the chart review noted appropriate steps taken by the clinical staff to explore this; thus, a retrospective review of the data places a limitation. For the patients that were transferred to a tertiary pediatric care facility with a neurosurgical department, our team was unable to capture the overall outcome with the management plan and length of stay at the tertiary center, as this is a limitation of this report. All children in this study had good clinical outcomes. Therefore, inpatient observation for children with isolated skull fractures and normal neurological examinations may suffice without being transferred or admission awaiting follow-up with consultation service. It is important to develop protocols to guide when planning which patients require hospitalization or transfer for a higher level of care at a tertiary care center.

## Conclusions

This limited dataset suggests that isolated, linear, non-displaced skull fractures with intact neurologic examination in children are at low risk for complications. This raises the question of whether these children need to be transferred to a pediatric trauma center or could be safely monitored in the primary non-pediatric trauma center where they are first seen. We believe clinically stable patients with no underlying brain injury on CT scan do not need to be admitted or transferred to a tertiary care center and can be observed safely in a non-pediatric neurosurgical center, provided there are no additional injuries. Multicenter studies are required to make a uniform recommendation and change practice, but this limited data suggests that children with isolated, linear, non-displaced skull fractures can be discharged safely from the ED after a brief period of observation.
